# Evaluation of length of central venous catheter inserted via cubital route in Indian patients

**DOI:** 10.4103/0972-5229.76081

**Published:** 2010

**Authors:** Saurabh Joshi, Anita Kulkarni, A. K. Bhargava

**Affiliations:** **From:** Department of Anesthesiology, Rajiv Gandhi Cancer Institute and Research Centre, Rohini, New Delhi, India

**Keywords:** Carina, central venous catheter, optimal length, peripherally inserted central venous catheter, radiopaque

## Abstract

**Aim:**

Peripherally inserted central venous catheters (PICCs) are popular due to the ease of insertion, low cost and low risk of complications. Anteroposterior (AP) chest radiograph (CXR) is then obtained to assess the location of the catheter tip. But poor-quality X-rays remain a significant problem. We planned a study using radiopaque marker at sternal angle, as a radiological landmark, to relate height of the patient and optimal length of PICC fixation, at the antecubital fossa, and to know the incidence of malpositioning.

**Materials and Methods:**

A total of 200 patients aged above 20 years, scheduled for elective major cancer surgeries were studied. Vygoflex PUR, 16-G catheter, length 70 cm was used. The right or the left arm was chosen depending on the availability of veins. Catheter tip was observed in the post procedure CXR.

**Results:**

200 patients [100 patients in group 1 (length of catheter fixation at antecubital fossa 45 cm) and 100 patients in group 2 (length of catheter fixation 50 cm)] were enrolled. The groups were further subdivided into 1a, 1b, 2a, 2b and results tabulated.

**Conclusions:**

Appropriate length of catheter fixation for group 1a was <45 cm, group 1b = 45 cm, group 2a = 50 cm, and for group 2b it was ≥50 cm. Gender and arm (right or left) did not have any bearing on the length of fixation. Incidence of malpositioning (15.5%) was more in right-sided catheters, more so, in short heighted people. PICC insertion via cubital route stands better compared with other routes, viz., Internal jugular vein IJV, subclavian and femoral.

## Introduction

Central venous catheter placement is often performed in clinical practice for the purpose of monitoring central venous pressure (CVP) to guide perioperative fluid replacement, administration of vasoactive drugs and rapid fluid administration. Peripherally inserted central venous catheters (PICCs) are especially useful for short-term use and in patients undergoing certain neurosurgical, otolaryngologic, oncosurgical and other major surgical procedures involving massive fluid shifts, or when free access to neck and clavicle is either not possible[1] or not desirable. PICC also avoids the physical discomfort and psychological distress associated with Trendelenburg position.[1]

Recent studies have shown that the right tracheobronchial angle or carina is a reliable anatomical landmark for the correct placement of central venous catheters.[2] A practical problem lies in identifying carina or right tracheobronchial angle in poor-quality chest radiographs. Malpositioning or suboptimal positioning can lead to complications like thrombosis, phlebitis, infections, accidental dislodgement, and catheter migration and catastrophic complications like cardiac tamponade.[3–13]

Various methods such as anatomical landmarks,[14,15] simple formulae,[16] right atrial electrocardiography[17–19] and echocardiography[20] have been used to ensure correct placement of the CVC tip. We planned a study with 200 patients using radiopaque marker at sternal angle as a radiological landmark to confirm the tip location of PICC, to evaluate the relationship between height of the patient and length of fixation of PICC at the antecubital fossa, and to know the complication rates.

## Materials and Methods

After getting approval from the institutional ethics committee, 200 patients aged above 20 years, scheduled for elective major cancer surgery, were studied. Vygoflex PUR, 16-G catheter, length 70 cm, was used for cannulation. The catheter was fixed at 45 cm for patients with a height between 141 and 160 cm (groups 1a and 1b) and at 50 cm for patients of height between 161 and 180 cm (groups 2a and 2b), viz., 1a = 141–150 cm, 1b = 151–160 cm (length of fixation at antecubital fossa is 45 cm); 2a = 161–170 cm, 2b = 171–180 cm (length of fixation at antecubital fossa is 50 cm).

The right or the left arm was chosen depending upon the availability of veins. All catheters were placed blindly. A check chest X-ray of the patient was done in the immediate postoperative period with the patient in supine position, radiopaque marker placed horizontally at sternal angle, and arms abducted and placed by the side of the patient.

The location of the tip was confirmed on chest X-ray. All the catheters were inserted ± 2 cm from the crease on the front of the elbow.

**Figure 1:**
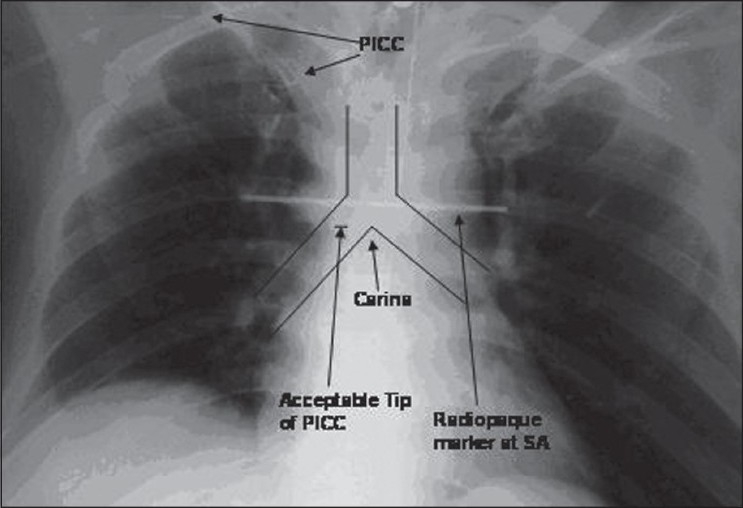
Acceptable (optimal) catheter tip

**Figure 2:**
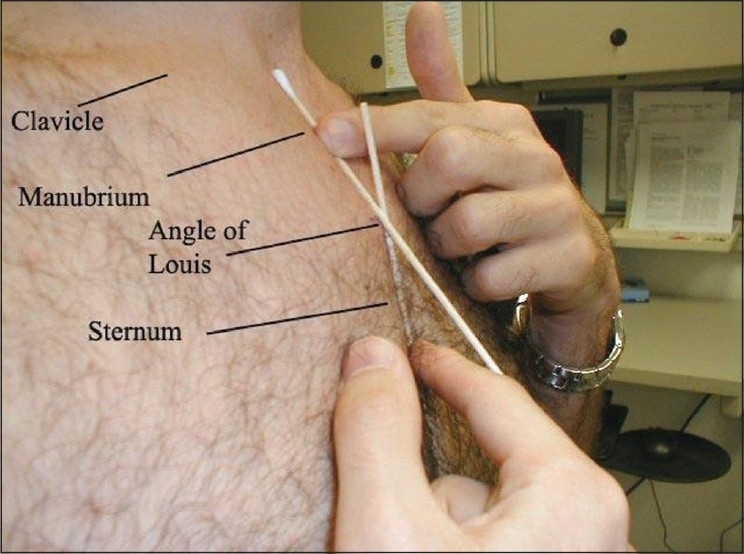
Site of placement of radiopaque marker (angle of Louis/sternal angle) marker not shown

**Figure 3:**
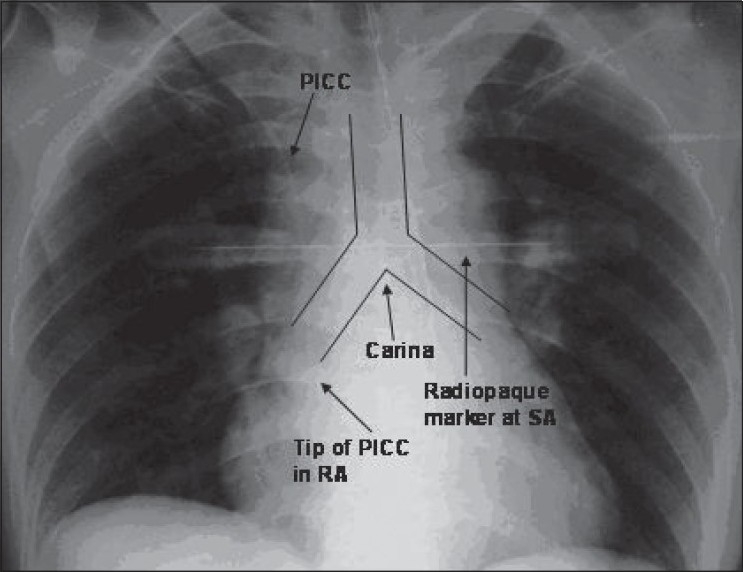
Non-acceptable (suboptimal) catheter tip

- *Optimal/acceptable* position was taken to be within 3 cm above and below the sternal angle/radiopaque marker [Figures 1 and 2].
- All the tip positions lying more than 3 cm above or below the sternal angle were taken as *suboptimal*.
- Coiled catheters or catheters with their tip reaching opposite sides were recorded as *malpositioned*.
- *Suboptimal + malpositioned* catheters taken *together* constitute *non-acceptable* catheter tips [Figure 3].

The outcome parameters, i.e., placement of catheter, location of the tip of PICC and malpositioning were statistically analyzed using chi-square test and binomial tests. Significance was taken at a *P* value of <0.05.

## Results

1. The mean height in group 1 was 154.26 ± 4.86 cm and in group 2 was 171.87 ± 5.84 cm.
2. Most of the patients in group 1 were found to be females (89%) and in group 2 males (94%) dominated. 46% of the PICCs in group 1 and 65% in group 2 were acceptable.
3. The acceptability of tip of catheter fixed at 45 cm (group 1) increased with increase in height (24.2% in group 1a and 56.7% in group 1b) up to 160 cm and this increase in optimal positioning was significant statistically (*P* = 0.002) as derived from the chi-square test.
4. The acceptability of tip of catheter fixed at 50 cm (group 2) increased with increase in height (58.3% in group 2a and 71.2% in group 2b) but this increase in optimal positioning was not statistically significant (*P* = 0.179), showing the adequacy of this catheter length for both these subgroups.
5. For various heights, non-acceptable tip positions were further analyzed for suboptimal or malpositioned tips [Table 1 and 2].
6. The acceptability of catheter tip was not significantly different for various height groups, when compared from left and right sides (*P* > 0.05), showing no influence of side/arm chosen on tip positioning [Table 3].
7. Total number of catheters inserted from the right side was 148 (74%) and from the left side were 52 (26%). Thirty-one (21%) of the malpositioned catheters were right sided, whereas none of the catheters from the left side was malpositioned (*P* = 0.000).
8. The incidence of malpositioning decreased from 30.3 to 1.9% with an increase in height from 141-150 to 171-180 cm, respectively. Ipsilateral IJV was the most common site for malpositioned PICCs (15 of 31), ipsilateral subclavian vein being the next common site (6 of 31). Some PICCs reached the contra lateral subclavian (3 of 31). Other sites were ipsilateral axillary vein (5 of 31) and contra lateral brachiocephalic vein (2 of 31).
9. Most of the malpositioning was in group 1 (22 of 31) and all the malpositioned catheters were right sided (31 of 31).

**Table 1:** Summarized results for catheter fixation in various groups

Group (height)	Optimal catheter length (cm)	Remark for catheter tip
1a (141–150 cm)	<45	Tip tends to lie below sternal angle
1b (151–160 cm)	45	Optimal tip position
2a (161–170 cm)	50	Optimal tip position
2b (171–180 cm)	≥50	Tip tends to lie above sternal angle

**Table 2:** Height vs. tip positions for all subgroups

Height	Acceptable tips	Non–acceptable (malpositioned) (%)	Non–acceptable (suboptimal)	Total (%)
1a (141–150 cm)	8	10 (30.3)	15	33 (100)
1b (151–160 cm)	38	12 (17.9)	17	67 (100)
2a (161–170 cm)	28	8 (16.7)	12	48 (100)
2b (171–180 cm)	37	1 (1.9)	14	52 (100)

**Table 3:** Comparative analysis of tip positions for catheters placed from right and left sides

Left		Height in cm (group)	Right		*P* value of acceptability from left and right sides
Acceptable (%)	Non-acceptable (%)		Acceptable (%)	Non-acceptable (%)	
1 (25.00)	3 (75.00)	141–150 (1a)	7 (24.10)	22 (75.90)	0.99
15 (60.00)	10 (40.00)	151–160 (2a)	23 (54.80)	19 (45.20)	0.67
10 (76.90)	3 (23.10)	161–170 (1b)	18 (51.40)	17 (48.60)	0.11
8 (80.00)	2 (20.00)	171–180 (2b)	29 (69.00)	13 (31.00)	0.7

## Discussion

Central venous catheters are used to provide secure access to the central circulation for CVP monitoring, administration of drugs, fluid resuscitation and total parenteral nutrition. PICCs offer the advantages of placement under local anesthesia, a low risk of major hemorrhage, no risk of pneumothorax and a lower cost. The position of these catheter tips is important because incorrect catheter tip placement may be associated with complications such as arrhythmias, thrombosis, phlebitis and cardiac perforation.

Various radiographic landmarks have been used to help identify and define the cephalad and caudal boundaries of the Superior vena cava (SVC).[21] PICC tips within a distance of 30 mm above and 50 mm below the carina were considered to be in an acceptable central location. The rates of successful initial PICC tip placement quoted in literature vary from 44 to 99%.[22–27] In our study, it was 55.5% which is in accordance with previous studies.

Ryu *et al*. concluded that CVC tip can be reliably placed near the carina level.[15] Kim *et al*. concluded that using the right third intercostal space as an anatomic landmark allows positioning of the catheter tip in the SVC near to but not in the RA in children,[28] Aslamy and colleagues suggested that the right tracheobronchial angle level constitutes the most reliable radiographic landmark. However, the right tracheobronchial angle and carina is difficult to identify, particularly on limited quality anterior-posterior chest radiographs.[6]

Peres utilized patients’ height to develop formulas to predict the optimum length of the catheter to be inserted for right internal or external jugular catheters, right infraclavicular subclavian catheters and left external jugular catheters.[6]

An appropriate reference point for CVP measurement is the midpoint of the right atrium which anatomically lies, on average, a vertical distance of 5 cm under the sternal angle or angle of Louis, where the second rib meets the sternum and creates a bump on the sternum.[29] Other evidences indicate that the trachea ends at its bifurcation at the level of the angle of Louis.[30] It also marks approximately the beginning and end of the aortic arch, and the bifurcation of the trachea into the left and right main bronchi (carina) and the level of the intervertebral disc between T4 and T5.[14,31–33]

In our study with sternal angle as the radiological landmark, height of the patient was found to correlate with length of catheter fixation. The puncture site was restricted to 2 cm above or below the cubital crease. Two predetermined lengths of catheter fixation were used, viz., 45 and 50 cm for heights greater than and lesser than 160 cm, respectively.

Venkatesan *et al*. found that though a central location was the most common tip position for right-sided PICCs, the brachiocephalic vein (ipsilateral) was the commonest location for left-sided ones.[34] In contrast, our study showed that acceptable positioning of catheter tips was not statistically different for right- and left-sided catheters.

Venkatesan *et al*. reported 11% of PICC entering the IJV.[34] An 18% incidence has been reported in two different studies by Ragasa and coworkers and Burgess and colleagues.[22,35] The same incidence quoted in pediatric population ranges from 14 to 37%.[36,37]

In the last group 2b, most of the catheters’ tips lied in upper part of the optimal spectrum, i.e., within 3 cm above the sternal angle, and also, most of the suboptimal catheters were actually falling short of the desired upper limit of the optimal range. So, probably a further 20–25 mm increase in length of catheter fixation would further increase the number of acceptable observations. This needs to be studied.

In our study, all the catheters were inserted while monitoring ECG. As soon as the ventricular ectopics were noted, the catheters were withdrawn by about 2 cm (and the cases were excluded from study). There were five such cases. This resulted in return of normal sinus rhythm and no medications were required for the control of the arrhythmias. All the patients were monitored perioperatively for arrhythmias and no significant observations could be made.

Our study shows that sternal angle is a reliable anatomic landmark, easily identifiable (with the help of radiopaque marker placed on it), on a poor-quality chest radiograph (where carina is not visible clearly), and can be used for optimal positioning of PICC tip. The length of fixation of PICC correlates well with the height of the Indian patient.

## Conclusions

This study shows that length of catheter fixation correlates well with height of the patient as follows.

1. 141–150 cm (group 1a): catheter length should be less than 45 cm.
2. 151–160 cm (group 1b): catheter length should be 45 cm.
3. 161–170 cm (group 2a): catheter length should be 50 cm.
4. 171–180 cm (group 2b): catheter length should be ≥50 cm.
5. The arm chosen does influence the optimal positioning of PICC tip but not in a manner which is statistically significant (*P* ≠ 0.05).
6. 55.5% of the catheter tip placements were acceptable.
7. The incidence of malpositioning of catheter tips is 15.5%.
8. Right-sided PICCs are more prone to malpositioning, predominantly in short heighted people, the most common site of malpositioning being ipsilateral IJV.
